# Screening of voice and vocal tract changes in professional wind instrument players

**DOI:** 10.1007/s00405-024-08755-1

**Published:** 2024-07-08

**Authors:** Ahmed M. El-Demerdash, Nirvana G. Hafez, Hanaa N. Tanyous, Kerollos M. Rezk, Mariam S. Shadi

**Affiliations:** 1 Phoniatrics Center, Military Medical Academy, Cairo, Egypt; 2 Department of Otorhinolaryngology, Phoniatrics Unit, Faculty of Medicine, Ain Shams University, Cairo, Egypt; 3 Higher Institute of the Conservatory, Academy of Arts, Cairo, Egypt

**Keywords:** Wind instruments, Vocal symptoms, VHI-10, Auditory-perceptual assessment, Laryngeal endoscopy, Pharyngeocele

## Abstract

**Purpose:**

Playing wind instruments is a strenuous task on the larynx, predisposing players to voice disorders. This study aims to evaluate potential vocal symptoms and vocal tract alterations in professional wind instrumentalists.

**Methods:**

In this cross-sectional study, 26 male military subjects were interviewed, completed the voice handicap index (VHI) -10 questionnaire, and subjected to auditory-perceptual assessment, neck examination, rigid laryngostroboscopy and flexible nasofiberoscopy both before and during instrument playing.

**Results:**

All participants had vocal fatigue symptoms, around one-quarter complained of voice change, one-quarter complained of shortness of breath while or after performing, and one-third complained of neck symptoms. The average score of VHI-10 was 16.2 ± 6.5, and approximately three-quarters of participants scored above the cut-off point. There were no significant correlations between age, years of instrument playing, average hours of daily practice, and VHI-10. Participants with neck symptoms had significantly higher VHI-10 scores. Those (around one-fifth) with an external neck swelling during Valsalva maneuver had a significantly higher VHI-10 score. Dysphonia, mainly mild and of strained, leaky quality, was detected in almost one-third of participants. While the instrument was being played, the vocal folds were somewhat adducted, and the vocal tract became more compressed as the task became more demanding. The most frequent observations in the vocal tract examination were hyperemia of the vocal folds or all over the laryngeal and pharyngeal mucosa, excessive secretions over the vocal folds, signs of hyperadduction, arytenoid edema, and phonatory waste.

**Conclusion:**

Wind instrumentalists frequently experience voice disorders, which necessitate further care and investigation.

## Background

Wind instruments constitute a wide variety of musical instruments and generate sound from the vibrations of blown air. They include both brass and woodwinds. Brass instruments are lip-vibrated; sound is created by the direct vibration of the lips on the metal mouthpiece. In woodwind instruments, on the other hand, sound is produced by the blown pulmonary airstream passing through the mouthpiece, which causes the reed(s) in the mouthpiece to vibrate [1].

Contrary to what was historically thought, the larynx does not merely open to allow the passage of pulmonary air but rather actively participates in sound production of wind instruments [1–3]; a narrowed glottis regulates the airflow of the “blow”. The embouchure (the position and use of the lips, tongue, and jaw in playing a wind instrument) may serve as an airflow receiver rather than controlling movement for “blow” [4]. Studies visualizing the larynx when playing wind instruments showed that playing musical tones was made possible when players blew with medialized vocal folds. Vocal folds do not entirely close but remain in an intermediate position [1, 2, 5] and vibrato was created by rhythmic glottal opening and constriction [4].

Since wind instrument musicians utilize their vocal tract extensively, they are at a higher risk of developing vocal damage as a consequence of performing on their instruments. Therefore, they have been recognized as professional voice users [6]. Voice problems significantly interfere with both the quality of life as well as the professional career of these musicians [1]. Nevertheless, only a few studies focused on voice disorders among wind instrumentalists [2]. On the other hand, the WHO have not listed voice disorders as a potential occupationally-related condition [7, 8]. Large epidemiological population-based research focusing on the health and financial repercussions of occupational voice problems are thus needed, so that these problems are adequately recognized [7].

Efficient sound production during the execution of wind instruments, especially for longer musical notes, requires deliberate and precise coordination of the orofacial and neck muscles’ position and tension, vocal tract configuration, diaphragmatic support, blowing pressure, pulmonary functions, mucociliary function, and humidification of air passages [4, 9–11].

In addition to laryngeal and voice problems [4, 9, 10], a range of occupational health hazards have been associated with playing wind instruments, whether in musical or medical literature. These include playing related musculoskeletal disorders (PRMDs) [12], temporomandibular disorders [13], malocclusion [14], hearing impairment [15, 16], focal task-specific oromandibular dystonia of the embouchure [17]. Other reported ailments that were attributed to increased intraoral pressure include increased intraocular pressure and intermittent high-pressure glaucoma [15, 18], pneumoparotitis [1, 4, 15, 19], hemoptysis [19], velopharyngeal insufficiency [19, 20], patulous eustachian tube [21], and laryngocele [19].

The aim of this work was to identify possible vocal symptoms and vocal tract changes in professional wind instrumentalists, as well as their perception of impairment due to such voice problems.

## Subjects and methods

This cross-sectional study invited 40 male wind instrument musicians of a military band in the Armed Forces, of whom 26 met the study’s selection criteria and were included. The study was conducted in the period between May 2022 and July 2023 at the Units of Phoniatrics of Ain Shams University and the Military Medical Academy. Included participants should have been playing wind instruments professionally for at least 3 years, aged above 20 years and below 50 years, are non-smokers, had no self-reported history of voice disorders, laryngeal diseases, pulmonary diseases, asthma, repeated upper respiratory tract infections, allergies, recent laryngeal intubation (within the past year), or medical conditions that may cause long-term straining e.g., constipation, chronic cough. Aside from their work, they should not engage in any other voice abusive or misusive behaviors, nor should they practice regular weightlifting exercises. All participants agreed to volunteer by giving their written informed consents, and all procedures were in accordance with the ethical standards laid down in the Declaration of Helsinki and were approved by the Research Ethics Committee at the Faculty of Medicine, (institute blinded for peer review).

Participants were interviewed to verify the selection criteria and gather demographic, professional, and medical data. Collected data included age, sex, the type of instrument played, the number of years of instrument playing experience, and the average number of hours of daily practice. History of any symptoms or diagnoses that are possibly related to playing wind instruments, such as velopharyngeal incompetency, hemoptysis, pneumoparotid, increased intraocular pressure, hypertension, hernia, varicocele, hemorrhoids, or laryngocele, was also noted. Participants also completed the voice handicap index-10 (VHI-10) [22] without assistance. They were asked to rate on a five-point scale ten statements that can describe their voices, vocal symptoms, and their effects on their lives, where 0 = never, 1 = almost never, 2 = sometimes, 3 = almost always, and 4 = always.

As participants were interviewed, their reported vocal complaints were analyzed, before the examination of their voices through auditory-perceptual assessment (APA). Subjects’ voices were recorded as they were asked to use a comfortable pitch and loudness to read a standardized text, count to ten, and sustain vowel prolongations, including /a/, /i/, and /u/ vowels. Subjects were asked to use a comfortable pitch and loudness and follow the protocol for standard voice recording which include reading a standardized text, counting to ten, and sustaining vowel prolongations. The recorded material was later independently rated by two experienced phoniatricians, who were blind to each other’s scoring, using the modified GRBAS scale [23]. In addition, the presence of hypernasality or other resonance disturbances in the participants’ speech was also noted.

The rating of voice samples included the overall perceived degree of dysphonia, if any, which is scored on a four-point scale where 0 is normal, 1 is slight, 2 is moderate, and 3 is severe. In the case of dysphonia, it describes the voice character/quality, as strained, leaky, breathy, or rough (irregular), and each character was graded in terms of severity as I, II, and III. An abnormal pitch (e.g., overall increased, decreased, or diplophonic), loudness (e.g., excessively loud, excessively soft, or fluctuating), or register (e.g., tendency of vocal fry at the end of a phrase or register break) were also commented on.

Following that, the neck was examined both at rest and upon performing a Valsalva maneuver. The larynx was then examined, first by an indirect rigid laryngoscope (Karl Storz 8706CA Hopkins 70 degree) for better visibility of the laryngeal structures and to assess the mucosal wave vibrations over the vocal folds using stroboscopy, and then by a flexible nasoendoscope (Karl Storz 11101RP2) to view the base of the tongue, pharynx, and larynx. A regular nasoendoscopic examination was done without instrument playing, and then the musician played two musical pieces, an easy one and a technically challenging one (judged by the third author according to the technical demands, rhythmic complexity, tonal challenges, articulation and phrasing), while being examined. All exams were done without topical anesthesia and were recorded in video and analyzed later by two experienced phoniatricians independently.

Any changes in the integrity of laryngeal or pharyngeal structures, masses/lesions, edema, hyperemia, glottal gaps during phonation, or excessive secretions were observed. Examination of the stroboscopic mucosal waves over the vocal folds as well as looking for signs of muscle tension dysphonia, e.g., side-to-side or anteroposterior constriction, also took place. The base of the tongue was examined for vascular lesions, masses, and any other abnormalities. In addition, as the subjects start to play their instruments while being examined, the position of vocal folds and vocal tract changes while the instrument is being played were noted.

The Statistical Package of Social Sciences (SPSS) (version 28) was used to analyze the results. The normality of the data was tested using the Kolmogorov-Smirnov single-sample test. Qualitative data were described as numbers and percents. Numerical variables were presented as mean and standard deviation (SD) or median and range. To compare the medians for the two categories of a dichotomous qualitative variable, the Mann-Whitney test was used. Spearman correlation was used to correlate continuous data. A *P* value ≤ 0.05 was considered significant.

## Results

The study included 26 male wind-instrument musicians. Their mean age (in years) was (mean 28.7, SD 7.7; range 21–50). The average instrument playing experience (in years) was (mean 10.2, SD 7.1; range 3–30), and the average daily practice (in hours) was (mean 3.4, SD 1.1; range 2–5).

The majority of the musicians used wood instruments (57.6%, *n* = 15) [this group consisted of 3 clarinet, 3 ney, 2 alto saxophone, 1 piccolo, 1 kawala, 1 oboe, 1 bagpipes, 1 fagot, 1 mizmar, and 1 baritone saxophone players], and the remaining played brass instruments (42.3%, *n* = 11); [this group consisted of 3 bugle, 2 trombone, 1 contrabass tuba, 1 flugelhorn, 1 cornette, 1 corno, 1 trumpet, and 1 euphonium players].

None of the participants complained of a history of hemoptysis, nasal regurge of food and/or fluids, or symptoms of increased intraocular pressure. Only one participant (3.8%) was known to be hypertensive. Three (11.5%) participants reported a history of hernia, one (3.8%) had a varicocele, and one (3.8%) complained of hemorrhoids.

All participants (100%, *n* = 26) had vocal fatigue/phonasthenia symptoms, 23.1% (*n* = 6) complained of a change in their habitual voice, and 26.9% (*n* = 7) complained of shortness of breath while performing (*n* = 4) or afterwards (*n* = 3). Around one-third of the participants (34.6%, *n* = 9) complained of one or more of the following neck symptoms, either during or right after playing their instrument: neck pain (*n* = 6), neck vein engorgement (*n* = 3), and neck fullness or swelling (*n* = 5). Other reported symptoms include coughing (*n* = 2), gastric pain (*n* = 1) while playing the instrument, and drowsiness (*n* = 2) afterwards.

The average score of the VHI-10 was (mean 16.2, SD 6.5). The frequency of participants scoring above the cut-off point of the VHI-10 (> 11) was 73.1% (n=19). The distribution of the different responses to VHI-10 statements is shown in Table 1. No significant correlations between age, years of instrument playing, average hours of daily practice, and VHI-10 were found (*r* = − .222, *p* = .275; *r* = − .174, *p* = .396 and *r* = .122, *p* = .552 respectively). Participants with neck symptoms had a significantly higher VHI-10 total score than those without symptoms (20.6 ± 6.4 versus 14.2 ± 5.7, *p* = .030). On examination, an external neck swelling upon performing a Valsalva maneuver was noted in 5 (19.2%) participants, and these participants also had a significantly higher VHI-10 total score than those without such swellings (23.2 ± 4.6 versus 14.5 ± 5.9, *p* = .008).

**Table 1:** The distribution of the different responses to VHI-10 questions

VHI-10 statement	Never	Almost never	Sometimes	Almost always	Always
1. My voice makes it difficult for people to hear me	6 (23.1%)	11 (42.3%)	9 (34.6%)	0 (0%)	0 (0%)
2. People have difficulty understanding me in a noisy room	3 (11.5%)	10 (38.5%)	10 (38.5%)	3 (11.5%)	0 (0%)
3. My voice difficulties restrict my personal and social life	5 (19.2%)	11 (42.3%)	6 (23.1%)	4 (15.4%)	0 (0%)
4. I feel left out of the conversations because of my voice	6 (23.1%)	17 (65.4%)	3 (11.5%)	0 (0%)	0 (0%)
5. My voice problem causes me to lose income	5 (19.2%)	11 (42.3%)	8 (30.8%)	2 (7.7%)	0 (0%)
6. People ask, “What’s wrong with your voice?”	1 (3.8%)	2 (7.7%)	17 (65.4%)	6 (23.1%)	0 (0%)
7. I feel as though I have to strain to produce voice	0 (0%)	0 (0%)	15 (57.7%)	9 (34.6%)	2 (7.7%)
8. The clarity of my voice is unpredictable	0 (0%)	4 (15.4%)	10 (38.5%)	8 (30.8%)	4 (15.4%)
9. My voice problem upsets me	2 (7.7%)	11 (42.4%)	8 (30.8%)	4 (15.4%)	1 (3.8%)
10. My voice makes me feel handicapped	6 (23.1%)	8 (30.8%)	8 (30.8%)	4 (15.4%)	0 (0%)

The results of the auditory-perceptual assessment (APA) of the participants’ voice and speech are shown in Table 2.

**Table 2:** Results of the auditory-perceptual assessment (APA)

Auditory-perceptual assessment	Total (*n* = 26)
Voice	
Normal	18 (69.2%)
Dysphonia	8 (30.7%)
Grade of dysphonia (*n* = 8):	
I	7 (87.5%)
II	1 (12.5%)
Character of dysphonia (*n* = 8):	
Strained, leaky, irregular	4 (50%)
Strained, leaky	2 (25%)
Irregular	1 (12.5%)
Breathy	1 (12.5%)
Change in voice pitch and register (*n* = 8):	
No change	3 (37.5%)
Lower pitch	2 (25%)
Lower pitch, register breaks	1 (12.5%)
Vocal fry	1 (12.5%)
Higher pitch	1 (12.5%)
Change in voice loudness (*n* = 8):	
No change	4 (50%)
Excessively loud	1 (12.5%)
Excessively soft	3 (37.5%)
Resonance	
Normal	24 (96.15%)
Slight hypernasality	1 (3.84%)

The findings of the vocal tract examination were as follows: The vocal folds were freely mobile in 96.2% (*n* = 25) of subjects [one player demonstrated limited abduction of both vocal folds], edematous in 30.8% (*n* = 8), hyperemic/showed increased vascular markings in 88.5% (*n* = 23), covered with excessive secretions in 73% (*n* = 19), showed glottal waste during phonation in 53.3% (*n* = 14), and showed abnormal stroboscopic mucosal waves in 23% (*n* = 6, 3 showed increased amplitude of vibrations, 2 showed left-right asymmetry in phase, and 1 showed both). Fullness of both ventricular folds was observed in 30.8% of subjects (*n* = 8), and it enlarged in 2 of them while playing the instrument. Signs of hyperadduction were common, with 50% (*n* = 13) of the participants showing ventricular band hyperadduction during phonation and 42.3% (*n* = 11) of them showing anteroposterior laryngeal compression during phonation. The arytenoids were edematous in 57.7% (*n* = 15), asymmetrical (due to a unilateral arytenoid sublaxation) in one (3.8%) participant, and the interaryenoid area was thickened in 23% (*n* = 6) of the participants. As they played their instruments, the vocal folds were partly adducted in all subjects, changing their opening and constriction to control airflow for different notes. In addition, we noticed that the more technically challenging the pieces to play, the greater the side-to-side tension and constriction in the larynx and the vocal tract.

None of the participants showed inadequate velopharyngeal closure. Hypertrophy of the lymphoid tissue at the tongue base was detected in one (3.8%) participant. Generalized edema, hyperemia, and excessive secretions involving most of the pharyngeal and laryngeal areas were seen in 3.8% (*n* = 1), 76.9% (*n* = 20) and in 34.6% (*n* = 9) of participants, respectively. Pyriform fossa(e) fullness was observed in 23% (*n* = 6; right sided in 2 subjects and bilateral in 4), and it increased in size in 4 of them upon playing the instrument [one player, who also had bilateral external neck swellings upon performing a Valsalva maneuver, was further examined with a CT scan, and the diagnosis of lateral pharyngeal diverticula (pharyngoceles) was confirmed].

The two judges demonstrated a high degree of inter-rater agreement on APA and assessment of vocal tract changes, with a majority of unanimous comments between the two raters. In cases of disagreement, both raters engaged in structured discussions, shared their perspectives to address disputes, and collaborated to reach a consensus.

No statistically significant relationships were detected between the VHI-10 total score and different findings on laryngeal examination.

## Discussion

The act of playing a wind instrument is a rigorous task on the larynx, predisposing wind instrumentalists to voice disorders. Nevertheless, voice problems in this population were understudied. This study contributes to our understanding of this topic. Its purpose was to investigate wind instrumentalists’ vocal tract behavior while playing their instruments, in addition to any voice disorders, laryngeal and pharyngeal findings they may encounter and how these are affecting them.

### Demographic characteristics of participants

The current study included 26 male instrumental musicians with a mean age of 28.7 ± 7.7. They practiced for an average of 3.4 ± 1.1 h daily for 10.2 ± 7.1 years. All participants were male, reflecting the gender profile of the military band from which they were recruited. In general, men’s participation in bands and orchestras usually exceeds that of women [1]. Women wind instrumentalists were included in two earlier studies investigating voice disorders in this population [2, 9] yet the results were not separately analyzed for each gender [1].

Several authors reported that beginner wind instrumentalists show more frequent laryngeal symptoms as compared to experienced ones [4, 9, 10, 24, 25]. Longer hours of daily practice and intense use of instruments can also induce vocal and laryngeal symptoms [10]. Nevertheless, the years of instrument playing as well as the average hours of daily practice were not correlated to the VHI-10 score in our cohort.

Wind instrument musicians are frequently subjected to increased intra-thoracic and intra-abdominal pressure, and are thus more likely to develop certain health problems [15]. Although not fully investigated, a few of our participants reported having some of those conditions related to increased intra-abdominal pressure. In order to rule out possible contributors to higher intra-abdominal pressure other than wind instrument playing, subjects with weightlifting practice, chronic cough, or constipation were excluded.

### Vocal symptoms

The majority of participants were not essentially seeking medical advice for their voice problems, and two of those who were diagnosed with dysphonia using auditory-perceptual assessment were not complaining of voice change, which demonstrates these professionals’ lack of awareness of voice disorders, despite being at risk of developing them.

Vocal fatigue/phonasthenia symptoms may include vocal fatigue, voice failure, lump in the throat e.g., globus sensation, throat tightness, frequent throat clearing, throat dryness, soreness/burning, neck pain/tenderness or episodes of voice change [26]. When asked about vocal symptoms, all participants reported vocal fatigue, with approximately one-fourth reporting a voice alteration. These findings are consistent with prior studies. The main complaints reported by wind instrumentalists in one study [2] were throat secretion (in 8 subjects) and dysphonia after intense use of the instrument (in 5 subjects). Playing-related health problems were evaluated in a group of 98 undergraduate and postgraduate student musicians (only 43 were wind instrumentalists) via a self-reported online survey [16]. Twelve students (though the number of wind instrumentalists was not specified) complained of throat discomfort, four of playing-related tension, and five of pain. In a cross-sectional questionnaire-based study [9], the 37 wind instrumentalists included scored highly on the Vocal Tract Discomfort Scale (VTDS). The most common VTDS symptoms reported by players, in terms of both frequency and intensity of occurrence, were dryness, aching, irritability, and cervical muscular pain, as well as the frequency of occurrence of fatigue after playing. de lima Silva et al. [27] also applied VTDS to a sample of 117 professional and student wind instrumentalists and found that the most frequent and intense symptoms were sensitive throat and sore throat, and the most intense symptom reported after playing the instrument was dryness. A recent literature review [1] summarized the main vocal symptoms reported by these professionals as phlegm, sore throat, dry throat, vocal tract irritability, pain, weak voice, voice breaks, hoarseness, and vocal discomfort following prolonged playing. Likewise, a systematic review [10] reported that the main vocal fatigue symptoms in wind instrumentalists, or laryngeal symptoms as they referred to them, were dryness, sore throat, throat irritation, throat clearing, discomfort, and tension, and these may or may not coexist with vocal symptoms; mainly altered voice quality, hoarseness, or voice failures.

Contrary to the studies mentioned above, one study [11] found statistically significant higher F0 and HNR values and lower perturbation parameters (jitter and simmer values) in the study group than the control group. The authors hypothesized that such findings indicate enhanced control of the phonatory and respiratory systems due to prolonged instrument use. It is important to note, though, that neither vocal complaints nor vocal tract findings were assessed in that study, and that the VHI-10 values were greater in the study group, which would reflect the consequences of vocal dysfunction.

In our cohort of participants, around one-fourth suffered from shortness of breath during or right after instrument playing. This is opposite to the results of a similar study [9] where VTDS was applied and none of the 37 participating male wind instrumentalists reported breathlessness. A possible explanation for this discrepancy may be related to the more extended practice hours in our cohort. A cough that only happens while playing the instrument was a less frequent symptom reported by two participants. We hypothesize that the increased airflow and pressure might have triggered this cough reflex. de Lima Silva et al. [10] also listed cough as one possible complaint that can be associated with intense use of wind instruments.

### Voice handicap index

VHI evaluates the subject’s perception of the impact of his voice problem. Higher scores indicate that a voice problem has a more severe handicapping effect on the individual’s life. In this study, the average score of VHI-10 was high, and around three-quarters of participants scored above the cut-off point. The total score was significantly higher in the presence of neck symptoms. A higher VHI-10 score was also previously obtained in wind instrument musicians than in healthy controls who had never played a wind instrument, and the difference was statistically significant [11]. Voice problems in these professionals can dramatically influence their quality of life [9]; de Lima Silva et al. [10] found that several vocal tract discomfort symptoms had a negative correlation with the Voice-Related Quality of Life (V-RQOL) scores.

### Auditory-perceptual assessment

Auditory-perceptual assessment characterizes vocal quality and quantifies voice deviations [1, 2]. Dysphonia was detected in almost one-third of the participants. It was mainly of a slight degree and of a strained, leaky quality. These findings are in accordance with an earlier study [3] that evaluated 30 male military wind instrumentalists’ voices using the GRBASI scale, and found they had greater alterations in vocal tension and instability parameters than control group subjects (12 percussionists), indicating that the larynx was used with effort [3]. In our study, the changes in voice quality, pitch, register, and loudness would also reflect tension and instability.

A well-controlled flow of expired air through the mouth and a firm velopharyngeal closure were shown to be prerequisites for maximum air pressure containment in the oropharyngeal regions [28]. Air entering the nasal cavity via a loose seal and escaping through the nose can thus be detrimental and career-ending [20]. However, velopharyngeal inadequacy is a common occurrence in wind instrumentalists [20, 29], particularly in clarinetists, French hornists, and double-reed instrumentalists [25, 29], due to fatigue of the velopharyngeal musculature [28] and inability to sustain prolonged exposure to high levels (can range from 12 to 90 cm H_2_O [28]) of intraoral pressure, a condition that is sometimes referred to as stress velopharyngeal insufficiency [20] and is usually perceived as an audible grunting, rustling, or snort [28] sound that seems to originate in the back of the throat [25].

Earlier in the literature, Levine [21] referred to this “loss of seal” as palatal paresis or paralysis rather than velopharyngeal inadequacy and hypothesized that exposure to constant pressure may stretch the palate, creating a loss of muscle tone and/or nerve weakness. One study [5] reported that velopharyngeal closure was sufficient for two wind instrumentalists while playing saxophone in the presence of the flexible nasoendoscope. In the current study, none of the participants had a history of nasal regurge of food or fluids or an inadequate closure of the velopharyngeal valve on nasofiberoscopic examination, yet one of them exhibited slight hypernasal speech as determined by auditory-perceptual assessment. The finding that none of the participants had stress velopharyngeal insufficiency on nasofiberoscopic evaluation could be attributed to the rapid nature of this examination, as this condition is generally observed after 30 min of playing [20, 29].

Velopharyngeal inadequacy is thought to be a common occurrence in wind instrumentalists [20, 28], due to fatigue of the velopharyngeal musculature [29] and an inability to withstand prolonged exposure to high levels of intraoral pressure, a condition referred to as stress velopharyngeal incompetence [20]. Nevertheless, none of the twenty-six participants reported a history of nasal regurgitation nor had an inadequate velopharyngeal closure on endoscopy, despite one of them exhibiting a slightly hypernasal speech. This finding could be attributed to the rapidity of the nasofiberoscopic examination, whereas stress velopharyngeal incompetence typically manifests after 30 min of playing [20, 28]. Similarly, one study [5] reported that velopharyngeal closure was adequate in two saxophonists while playing with a flexible nasoendoscope present.

### Vocal tract examination

The vocal folds were partly adducted in all participants while the instrument was being played, and the vocal tract became more squeezed as the task got more demanding. These findings are unanimous with earlier observations [1, 2, 5].

The main vocal tract findings observed in the majority of our series were hyperemia of the vocal folds or all over the mucosal covering of most of the pharyngeal and laryngeal structures, excessive secretions over the vocal folds, signs of hyperadduction, arytenoid edema, and phonatory waste. Similarly, Eckley [2] found arytenoid and interarytenoid edema and hyperemia in 8 (moderate in 5, and mild in 3) of the 10 wind instrumentalists studied. These changes may indicate a non-organic voice disorder or signal inflammation.

Wind instrumentalists can develop laryngeal soft-tissue swellings and vocal fold lesions, especially if the player adopts laryngeal hyperfunction (tense glottic and supraglottic areas) in an attempt to compensate for air loss above in cases of velopharyngeal inadequacy [25]. Such lesions were not detected in our series, yet they can be expected to develop if voice disorders are left untreated. A longitudinal study would be helpful to investigate this speculation.

Pharyngoceles and laryngoceles are rare conditions. Their acquired type, among other causes, can be more frequently seen in individuals practicing activities that involve increased intrapharyngeal and intralaryngeal pressure for prolonged periods, such as blowing musical instruments [30]. They can be asymptomatic and are frequently misdiagnosed or interchangeably diagnosed [30]. Several players in this study showed fullness of the ventricular folds and/or the pyriform fossa(e) that increases in size as they start playing their instrument while we examine their vocal tract. This raises the index of suspicion of laryngoceles and pharyngoceles, especially in those with concomitant external neck swelling during Valsalva. Nevertheless, no definitive diagnosis through imaging during Valsalva was obtained except in one subject who agreed to undergo CT scanning, which confirmed the presence of bilateral pharyngoceles. On the other hand, the observed ventricular fullness was bilateral, while around 80–85% of the reported laryngocele cases were unilateral [31].

Abnormal stroboscopic mucosal waves were detected in nearly one-quarter of the subjects in the current study, in the form of increased amplitude of vibration, asymmetry in phase, or both. Left-right phase asymmetry is defined as a discrepancy in the phase of the vibratory cycle between the right and left vocal folds, that can be caused by differences in the mass and/or tension of the vocal folds [32]. The amplitude of vibration refers to the extent of lateral excursion of the mucosa covering each vocal fold during vibratory activity as viewed on videotroboscopy, i.e., the vocal fold’s medial edge displacement during the vibratory cycle from the maximum closed point to the maximum open point [34]. Following Bless et al.‘s [33] definition, the amplitude of vibration that is more than one-third the width of the visible part of the vocal folds was considered to be increased. Larger amplitudes of vibration occur with increased vocal folds’ pliability (as pliable tissues are easier to displace by airflow), increased subglottal pressure (whether by expanding the volume of expired air or intensifying medial compression of the vocal folds secondary to increased laryngeal muscular contraction) [34]. Vocal fold edema was prevalent among participants, and it alters the mechanical properties of the vocal fold mucosa, rendering it more pliable. Despite the increased mass from fluid accumulation, the tissue affected by edema might become more compliant due to the fluid’s presence, contributing to greater displacement during vibration and leading to an apparent increase in vibratory amplitude despite the increased mass. Moreover, subglottal pressure is increased secondary to blowing, and hyperadduction is possible, especially with a faulty technique.

### Pathophysiology of voice disorders in wind instrumentalists

While laryngeal use in wind instrumentalists differs from that in professional voice users, some possible pathophysiological explanations behind the development of voice disorders in wind instrumentalists exist. They can be attributed to the active engagement of the larynx, and the exhaustion associated with glottic constriction and opening to control airflow. The effort in the glottal region during vocal fold adduction may build muscular tension and subglottal pressure over time [1, 10], causing laryngeal strain and eventual vocal symptoms and laryngeal lesions [4, 5, 9]. de Lima Silva et al. [10] pointed out that since cervical pain is one of the most common complaints experienced by musicians and the intrinsic and extrinsic laryngeal muscules, located in the cervical region, are recruited when playing a wind instrument, complaints of vocal tract discomfort can be related to the overload effort of these muscles. Moreover, wind instrumentalists may improperly use their glottis, contributing to vocal damage, such as with forceful adduction of the vocal folds during performance [25] or the use of the Valsalva maneuver while playing their instruments.

Furthermore, wind instrumentalists often use oral breathing with quick deep inhalation and delayed exhalation [10]. It involves excessive flow of the expired air through the oral cavity and vocal tract, exerting high pressure on structures [1, 11] and favoring dryness [1, 9, 10]. Subsequently, tissues become less elastic and more liable to friction and mechanical trauma [10]. The lack of hydration and lubrication of the vocal folds, following oral breathing and dry mouth, increases the risk of vocal effort and dysfunction [9].

On the other hand, while all of these mechanisms can be linked to playing wind instruments, no conclusive etiology for voice problems has been established [25]. We assume that voice disorders in our series may be related to instrument use itself and/or to other causes that may predispose to vocal problems in general, e.g., other parallel vocal activities or poor voice hygiene. Other authors have previously proposed similar assumptions. de Lima Silva et al. [10] suggested that vocal and laryngeal symptoms in wind instrument players were associated with individual factors (previous voice change [10, 35], inappropriate vocal health habits [3], individual physical differences among instrumentalists [25], gastroesophageal reflux [27] and organizational factors (working hours, intensive use, instrument type, and technique). de Lima Silva et al. [27] concluded that vocal tract discomfort symptoms experienced by wind instrument players in their study are likely work-related because their perception changed after playing the instrument.

Further research is needed to find out if voice problems among wind instrumentalists are really avoidable with voice hygiene and proper technique. It is suggested that the glottal functioning dynamics during proper playing techniques of wind instruments do not necessarily traumatize the vocal folds’ free edges [4], and that instrumental performance misuses may be the cause of voice damage in wind instrumentalists. These musicians may benefit from vocal hygiene strategies such as adequate hydration [9] and proper training of less traumatic instrumental techniques [25]. A collaboration between medical professionals and expert musicians in research would be valuable.

### Clinical implications

The outcomes of this study can influence clinical practice in several ways. It can promote the awareness of healthcare professionals working with wind instrumentalists and urge them to advocate for healthy playing techniques, as well as routinely screen for voice problems in this population, in order to early identify and treat these vocal issues before they escalate. It can also enlighten wind instrument players towards practicing strategies to prevent or manage voice problems related to instrument playing. The results shall encourage further research into the specific mechanisms causing voice problems in wind instrumentalists and integrate this knowledge into educational programs for both musicians and healthcare providers.

### Limitations

This study tackles an understudied area, yet it has some limitations that should be taken into consideration while evaluating the results. The low number of participating volunteers did not allow for some analyses or adequate subgrouping of certain variables. The included musicians played a wide variety of wind instruments; therefore, conclusions about distinct groups of players based on the type of instrument could not be specifically drawn. Given the peculiarities of each instrument’s sound source, material, shape, acoustical properties, use of pulmonary airflow, etc., glottic configuration, vocal tract adjustments, effort needed for execution, and vocal symptoms may differ with each instrument type and technique [1, 10]. Besides, no control group was included for comparison, and all participants belonged to the same band. In addition, the discussion of our results was hampered by the modest number of published articles.

## Data Availability

The datasets used and analyzed during the current study are available from the corresponding author on reasonable request.
